# A Simple Alkaline Method for Decellularizing Human Amniotic Membrane for Cell Culture

**DOI:** 10.1371/journal.pone.0079632

**Published:** 2013-11-13

**Authors:** Mehrnoosh Saghizadeh, Michael A. Winkler, Andrei A. Kramerov, David M. Hemmati, Chantelle A. Ghiam, Slobodan D. Dimitrijevich, Dhruv Sareen, Loren Ornelas, Homayon Ghiasi, William J. Brunken, Ezra Maguen, Yaron S. Rabinowitz, Clive N. Svendsen, Katerina Jirsova, Alexander V. Ljubimov

**Affiliations:** 1 Eye Program, Cedars-Sinai Medical Center, Los Angeles, California, United States of America; 2 Regenerative Medicine Institute, Cedars-Sinai Medical Center, Los Angeles, California, United States of America; 3 Departments of Biomedical Sciences, Surgery, and Neurosurgery, Cedars-Sinai Medical Center, Los Angeles, California, United States of America; 4 University of California Los Angeles, Los Angeles, California, United States of America; 5 Department of Integrative Physiology, University of North Texas Health Science Center, Fort Worth, Texas, United States of America; 6 Departments of Ophthalmology and Cell Biology, State University of New York, Downstate Medical Center, Brooklyn, New York, New York, United States of America; 7 American Eye Institute, Los Angeles, California, United States of America; 8 Laboratory of the Biology and Pathology of the Eye, Institute of Inherited Metabolic Disorders, 1^st^ Faculty of Medicine and General Teaching Hospital, Charles University, Prague, Czech Republic; University of Illinois at Chicago, United States of America

## Abstract

Human amniotic membrane is a standard substratum used to culture limbal epithelial stem cells for transplantation to patients with limbal stem cell deficiency. Various methods were developed to decellularize amniotic membrane, because denuded membrane is poorly immunogenic and better supports repopulation by dissociated limbal epithelial cells. Amniotic membrane denuding usually involves treatment with EDTA and/or proteolytic enzymes; in many cases additional mechanical scraping is required. Although ensuring limbal cell proliferation, these methods are not standardized, require relatively long treatment times and can result in membrane damage. We propose to use 0.5 M NaOH to reliably remove amniotic cells from the membrane. This method was used before to lyse cells for DNA isolation and radioactivity counting. Gently rubbing a cotton swab soaked in NaOH over the epithelial side of amniotic membrane leads to nearly complete and easy removal of adherent cells in less than a minute. The denuded membrane is subsequently washed in a neutral buffer. Cell removal was more thorough and uniform than with EDTA, or EDTA plus mechanical scraping with an electric toothbrush, or n-heptanol plus EDTA treatment. NaOH-denuded amniotic membrane did not show any perforations compared with mechanical or thermolysin denuding, and showed excellent preservation of immunoreactivity for major basement membrane components including laminin α2, γ1-γ3 chains, α1/α2 and α6 type IV collagen chains, fibronectin, nidogen-2, and perlecan. Sodium hydroxide treatment was efficient with fresh or cryopreserved (10% dimethyl sulfoxide or 50% glycerol) amniotic membrane. The latter method is a common way of membrane storage for subsequent grafting in the European Union. NaOH-denuded amniotic membrane supported growth of human limbal epithelial cells, immortalized corneal epithelial cells, and induced pluripotent stem cells. This simple, fast and reliable method can be used to standardize decellularized amniotic membrane preparations for expansion of limbal stem cells *in vitro* before transplantation to patients.

## Introduction

Limbal epithelial stem cells (LESC) in the human cornea reside at its periphery known as the corneoscleral limbus and continuously renew the corneal epithelium [1]–[3]. In some conditions these cells degenerate and/or die, leading to limbal epithelial stem cell deficiency (LSCD). It is a fairly common and clinically important cause of corneal blindness. LSCD may develop as a consequence of congenital aniridia, Stevens-Johnson syndrome, chemical and thermal burns (including sulfur mustard gas poisoning in war conditions), ocular cicatricial pemphigoid, chronic inflammation, and microbial infections [4]–[6]. Ocular burns that frequently lead to LSCD comprise up to 18% of all eye injuries [7]. More recently, an increasing number of long-term contact lens wearers have been also diagnosed with LSCD [8]. LSCD results in corneal erosions and vascularization, conjunctival ingrowth (conjunctivalization), and scarring, causing compromised corneal transparency and gradual vision loss [4], [5]. This condition may be hard to treat especially in cases of total stem cell deficiency [4].

Transplantation of LESC cultured on human amniotic membrane (HAM) or fibrin to the affected limbal area has emerged as a promising approach to manage LSCD since the pioneering work of Tseng’s group [1], [2], [9], [10]. Although the procedure is not fully standardized [11] and allograft survival is low, even with immunosuppression, an average 1–3 years success rate of up to 76% was reported [1], [2], [9].

HAM continues to be the most popular substratum for LESC to expand and then use for transplantation purposes in LSCD patients. It is readily available from placenta discarded in delivery rooms, and has been a more successful alternative to previous methods, with hundreds of patients having received culture-expanded LESC transplants [12], [13]. Amniotic cell basement membrane (BM) is largely (but not completely) similar to limbal BM in composition [14], [15]. HAM contains important growth factors [16]–[18], is anti-bacterial, anti-angiogenic, only very weakly immunogenic [19]–[21], and improves wound healing [20], [22], [23]. Although LESC can differentiate on HAM [24], it is still considered the best substratum for their expansion, also allowing secure placement onto the patient’s cornea [11], [13].

HAM has been generally used for clinical purposes as intact (cryopreserved, lyophilized or dry) and denuded, after amniotic epithelial cells removal for better LESC adhesion. Intact HAM mostly supports the growth of limbal explants [24]–[26], whereas denuded HAM can be used as good scaffold for enzymatically dispersed LESC [16], [18], [27]–[30]. Denuded HAM better supports LESC proliferation, shows an increased preservation of clonogenicity, and is less immunogenic [16], [18], [20], [25], [26], [30], [31]. Various methods have been used to denude HAM including treatment with dispase, thermolysin, trypsin, EDTA, sodium dodecyl sulfate (SDS), ammonia or urea [16], [18], [27], [32]–[38]. Most such treatments last for hours, are tedious, may damage HAM or remove some components, fail to uniformly remove the epithelium, require additional scraping, and are not very reproducible [18], . The existing treatments also affect the stroma, which may result in the partial removal of HAM’s growth factors [18]. Realizing the need for a better approach, we took advantage of a long-known ability of alkaline solutions to dissolve cells [41], [42] and applied mild solutions of sodium hydroxide only to the amniotic epithelial cells for less than a minute as a fast and reproducible HAM denuding agent. The procedure was efficient in thoroughly removing amniotic epithelial cells from HAM. This fast and reproducible method using an inorganic agent could significantly streamline and standardize denuded HAM preparation for LESC culture.

## Results

**Figure 1:**
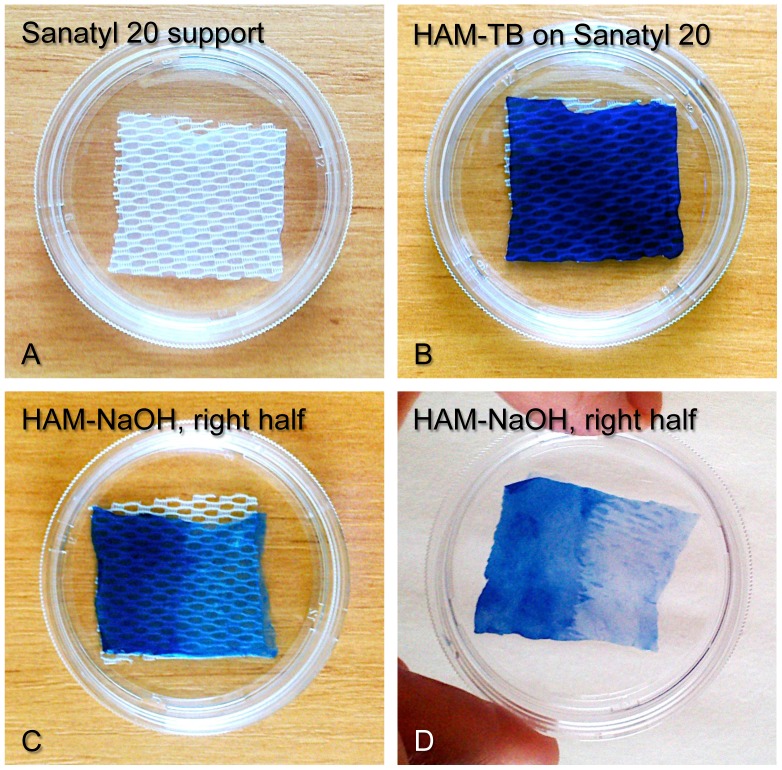
Glycerol-cryopreserved HAM after thawing, Trypan blue staining and treatment. A, mesh Sanatyl 20 support for HAM in a Petri dish. B, HAM on Sanatyl 20 after TB staining (the whole membrane is stained as glycerol kills the cells). C, the right half of HAM treated with NaOH (staining is significantly lighter than on untreated left half). D, the same HAM without Sanatyl 20 support; note lack of TB staining (cell removal) on the most part of the treated right half. Low-magnification pictures are presented.

**Figure 2:**
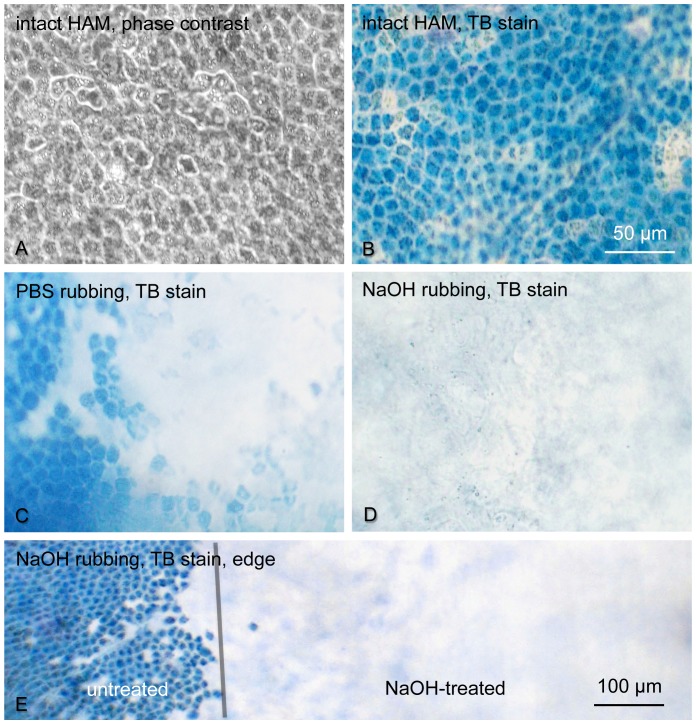
Cell removal by NaOH from cryopreserved HAM. A, phase contrast of cryopreserved and thawed out HAM showing a monolayer of amniotic epithelial cells. B, the same HAM stained with TB; all cells are stained. C, cotton swab rubbing of HAM leaves many amniotic epithelial cells behind. D, after 30 sec rub with 0.5 M NaOH-soaked cotton swab no cells are left. E, NaOH effectively removes cells from HAM (gray line shows the boundary of the rubbed zone). Bar in A–D = 50 µm; in E, bar = 100 µm.

The successful removal of amniotic epithelial cells from HAM with NaOH was obtained independently in two laboratories: one at Cedars-Sinai Medical Center (Los Angeles) and another at Charles University (Prague). Gentle rubbing of HAM with 0.25–0.5 M NaOH-soaked cotton tip for less than one minute removed most of the cells, as shown on Fig. 1. To better visualize the debridement, HAM was stained with Trypan blue (TB) that is excluded by live cells. Cryopreservation with glycerol effectively kills amniotic epithelial cells [43], resulting in a membrane that is entirely stained blue. Denuding the right half of the HAM piece with NaOH effectively removed the cells as evidenced by the loss of blue color (Fig. 1). At higher magnification, HAM presented a monolayer of tightly packed epithelial cells, all stained by TB (Fig. 2A,B). Rubbing it with a cotton tip soaked in PBS removed cells only partially, whereas rubbing with NaOH-soaked tip efficiently removed all cells judged by uniform loss of TB-stained material (Fig. 2C–E).

**Figure 3:**
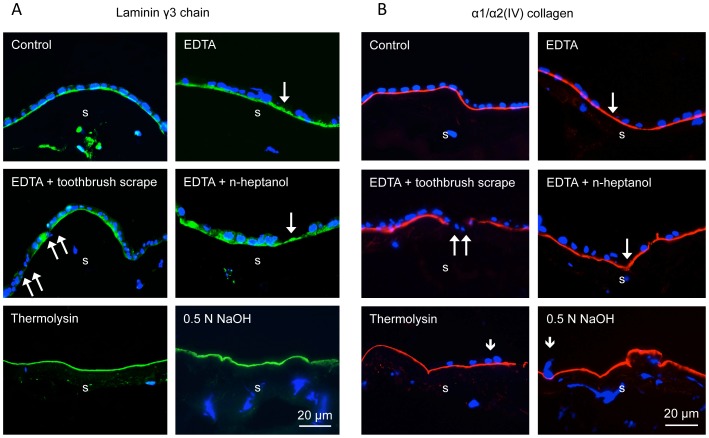
Laminin γ3 chain and type IV collagen α1/α2 chain expression in intact HAM and after various treatments. Laminin γ3 (A) and type IV collagen α1/α2 (B) are found in the limbal epithelial BM but not in the central corneal BM. Here and below, single long arrows show denuded parts with no cells (DAPI-counterstained nuclei). Double arrows show places where HAM is disrupted after scraping the membrane with electric toothbrush on low speed. EDTA together with rubbing or n-heptanol leaves a number of epithelial cells still attached to HAM. In contrast, thermolysin and NaOH leave little (single short arrows) to no epithelium on the treated HAM. Except for EDTA+toothbrush scraping, all treatments preserve normal continuous staining patterns of both BM components. Immunohistochemical staining of O.C.T.-embedded and sectioned HAM. Bar = 20 µm.

**Figure 4:**
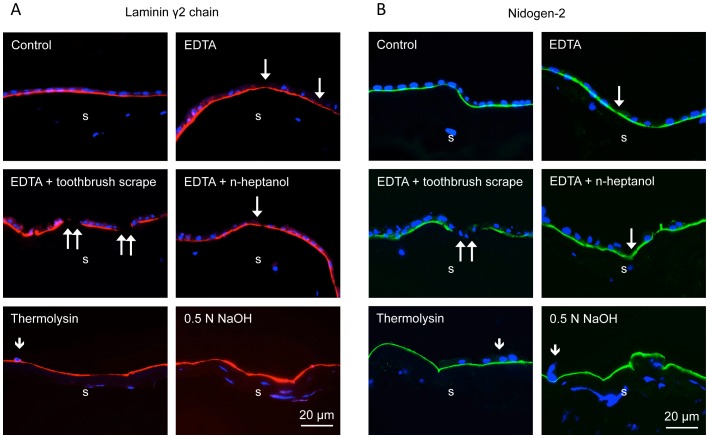
Laminin γ2 chain and nidogen-2 expression in intact HAM and after various treatments. Laminin γ2 (A) and nidogen-2 (B) are also expressed in central and limbal epithelial BM. The effects of various treatments are similar to the ones shown on Fig. 3. EDTA treatment results in incomplete cell removal or even HAM damage (double arrows) after extensive scraping. Thermolysin treatment removes cells well but shows some local irregular staining for proteolysis-sensitive laminin γ2 chain (A). NaOH produces HAM virtually devoid of epithelial cells with continuous staining for both BM components. Immunohistochemical staining of O.C.T.-embedded and sectioned HAM. Bar = 20 µm.

**Figure 5:**
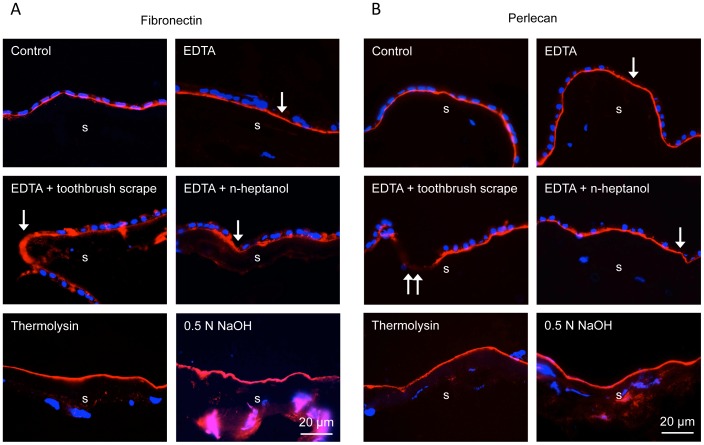
Fibronectin and perlecan expression in intact HAM and after various treatments. The results on fibronectin (A) and perlecan (B) are similar to other BM components. Only thermolysin and NaOH completely remove amniotic epithelium. Immunohistochemical staining of O.C.T.-embedded and sectioned HAM. Bar = 20 µm.

It was important to compare NaOH method with several others described before in terms of HAM integrity and expression of major BM components identified by immunofluorescent staining of 1% formalin-fixed cryostat sections. The BM components were chosen from those predominantly expressed in the limbal BM, such as laminin chains γ3 (Fig. 3A), α2, β2 (data not shown here), and collagen type IV α1/α2 chains (Fig. 3B), and those also expressed in central corneal BM, such as laminin γ2 chain (Fig. 4A), nidogen-2 (Fig. 4B), fibronectin (Fig. 5A), perlecan (Fig. 5B), laminin β1 and γ1 chains, and collagen type IV α6 chain (data not shown here). Of these components, laminin β1 and β2 chains could not be detected, whereas all others yielded a positive (although sometimes variable staining (e.g. for laminin α2 chain). As shown in Figs. 3–5, EDTA treatment did not compromise HAM integrity judged by continuous staining for all tested BM components. However, although this treatment removed some cells (single long arrows), many cells were generally left behind and persisted upon gentle scraping. More vigorous scraping with low-speed electric toothbrush caused local ruptures of HAM (double arrows on Figs. 3, 4, 5B). Additional rubbing with n-heptanol-soaked cotton applicator was helpful but still some cells remained. Brief thermolysin treatment efficiently removed most or all cells (few remaining ones are marked with, but HAM became fragile and was easily fragmented during O.C.T. embedding.

**Figure 6:**
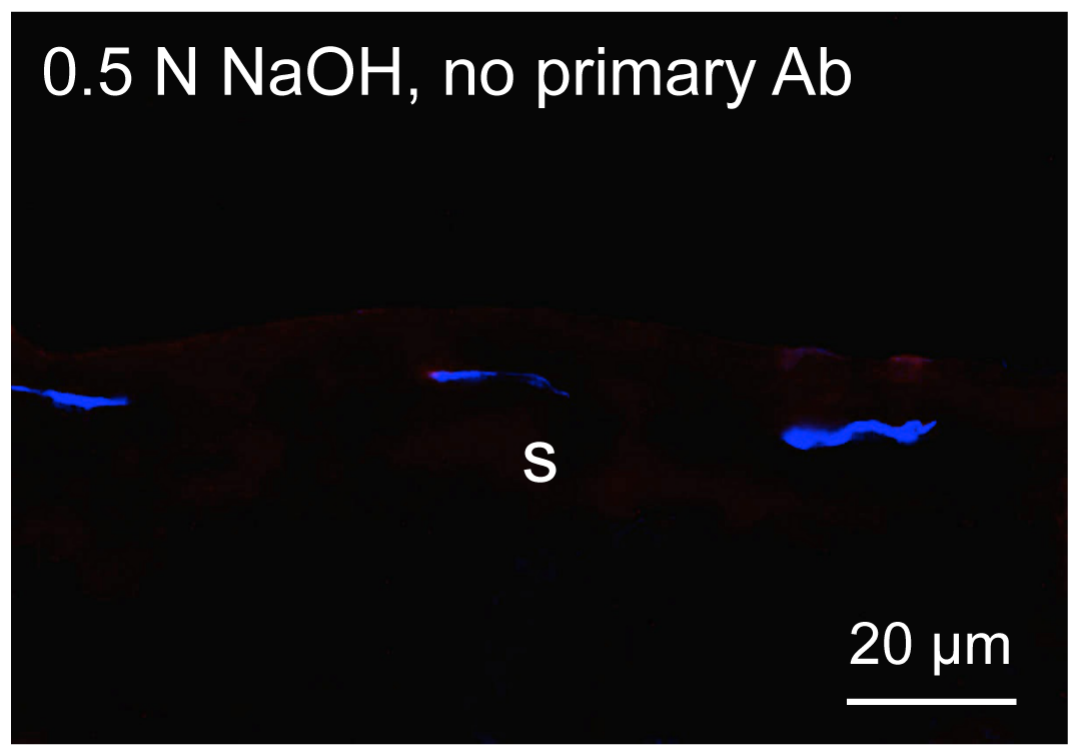
Negative control with omission of primary antibody. Immunohistochemical staining of OCT-embedded and sectioned HAM treated with NaOH. No staining is seen apart from DAPI nuclear staining of HAM stromal cells. Bar = 20 µm.

NaOH treatment was the only one that efficiently removed amniotic epithelium from HAM with only occasional cells remaining (short arrows; Figs. 3B and 4B), while at the same time preserving continuous staining for all BM components (Figs. 3–5). The negative controls showed that this staining was not an artifact due to the known “edge effect” (Fig. 6).

In initial experiments, we used 0.5 M solution for less than 30 sec rubbing that was very efficient in removing cells. Later, we also tried lower concentrations that were efficient down to 0.25 M; however, additional gentle rubbing was usually needed.

**Figure 7:**
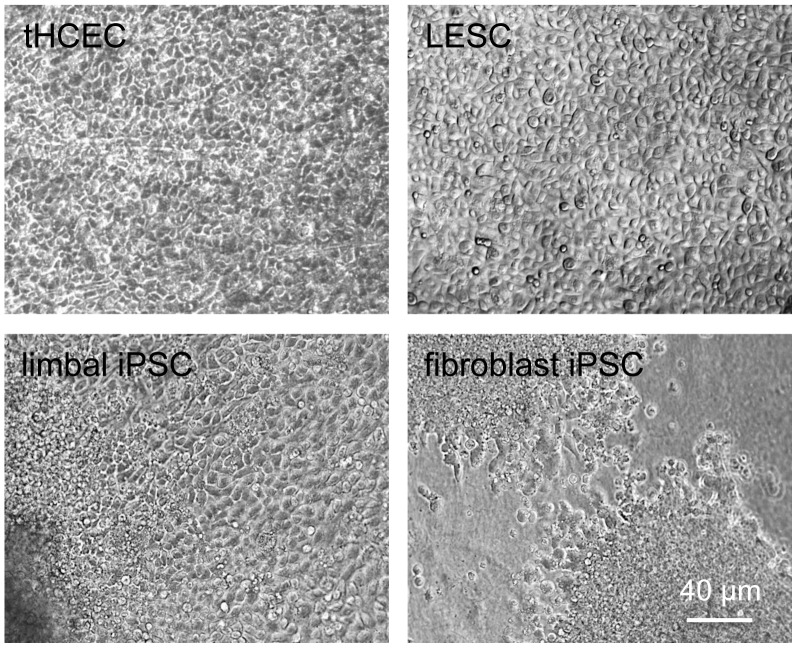
Phase contrast of various cells grown on NaOH-denuded HAM. Note cobblestone monolayer of telomerase-immortalized corneal epithelial cells (tHCEC), LESC, and LESC-derived iPSC. Fibroblast-derived iPSC also show good spreading of cells and growth. Bar = 40 µm.

**Figure 8:**
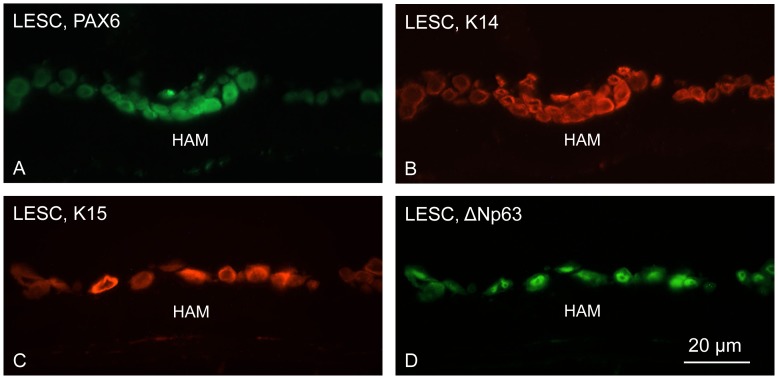
Marker analysis of LESC-enriched cultures grown on top of NaOH-denuded HAM. Note expected positive staining for putative LESC markers PAX6, K14 (A, B; double label), K15, and ΔNp63 (C, D; double label). Immunohistochemical staining of O.C.T.-embedded and sectioned HAM. Bar = 20 µm.

NaOH-denuded HAM was further used as culture substratum for human cells including telomerase-immortalized corneal epithelial cells (tHCEC), primary LESC, LESC-derived induced pluripotent stem cells (iPSC) or skin fibroblast-derived iPSC. NaOH-treated HAM supported proliferation of epithelial cells and iPSC well (Fig. 7). Cells formed confluent cobblestone monolayers within 1–2 weeks. The expression of some putative LESC markers was further evaluated in primary human LESC cultured on NaOH-denuded HAM. LESC expressed PAX6, keratins 14 and 15, as well as ΔNp63 (Fig. 8), similar to their expression reported in cultured cells and *ex vivo* corneas [26], [30], [44].

## Discussion

Since the initial successful graftings performed in 1960–1970, non-immunogenic HAM has been widely used as a biodegradable patch in corneal surgery and as natural scaffold for culturing LESC as a transplant for treatment of LSCD [10], [32], [45], [46]. The epithelium tightly covering HAM is a poorly adhesive scaffold for most if not all epithelial cells. If amniotic epithelial cells are not removed, LESC monolayers cannot be easily established on HAM. Only limbal explants can develop substantial outgrowths on intact HAM, possibly by displacing amniotic epithelial cells. Cryopreservation of HAM with a mild fixative glycerol that is allowed in the European Union [47] kills amniotic cells [43] allowing for an easier growth of explanted LESC-enriched limbal fragments. However, when compared to denuded HAM, intact membrane supports growth of cultured LESC less well and promotes terminal differentiation of these cells especially when the cultures are air-lifted [31], [48]. Therefore, it is preferable to use decellularized HAM in order to obtain LESC sheets best suitable for transplantation. At the same time, it may be important to keep stromal cells out of contact with denuding agent as much as possible, as they secrete factors needed to promote cell proliferation and wound healing [16], [17].

All existing techniques for denuding HAM with EDTA, dispase, trypsin, thermolysin, NH_4_OH, SDS or urea [18], [27], [31], [36], [38], [39], [49]–[52] require soaking HAM in cell stripping solutions, from 15 min to 24 hrs, which may impact both its epithelial and stromal compartments. Additionally some drawbacks have been associated with most denuding methods, necessitating further standardization of this important procedure. For instance, dispase routinely used to isolate LESC, and to a certain extent trypsin cause HAM destruction [18], [27], [39]. EDTA even with overnight incubation does not remove amniotic epithelium completely and additional scraping is required [27], [29]. This was also confirmed in our experiments, and EDTA was even reported to cause some damage to HAM [39]. Applying an electric toothbrush at low speed after EDTA resulted in HAM tears in our study (Figs. 3–5). Additional post-EDTA brief rubbing with n-heptanol used to remove corneal epithelial cells for wound healing studies [53] still left some epithelial cells attached. Thermolysin and 5 M urea denude HAM well but some destruction of laminin was noted [18], [39]. In our experiments, thermolysin efficiently removed amniotic epithelial cells but the membranes became fragile and hard to handle. However, after cryopreservation with a mild fixative glycerol, thermolysin was reported to cause no damage to HAM [40]. SDS procedure is very long (up to 48 hrs), involves nuclease treatment and the preservation of HAM components has not been analyzed [16], [30], [36].

Therefore, we set out to design a fast and reliable method for denuding HAM that would not require the incubation of the whole membrane in the decellularizing media and would allow for complete removal of the denuding agent. Mild alkali that are easy to neutralize and can readily solubilize cells [41], [42] could fit these tasks well. Previously, 10–15 min soaking in 0.1%–10% ammonium hydroxide with additional scraping has already been used to denude HAM [33], [49]–[52] for cell culture but this method has been largely abandoned. Consequently, we revisited the old alkaline cell solubilization technique applying faster acting NaOH rather than NH_4_OH to denude HAM. The advantages of our method are its speed, efficiency, and apparent lack of direct contact of cell stripping solution with the HAM stroma. The denuding agent is inorganic and is easily and immediately neutralized in PBS yielding just salt, which may make it attractive for regulatory agencies. Another important aspect of the NaOH method is that it is very efficient as well on HAM cryopreserved in glycerol (Figs. 1, 2), which is an officially recommended cryopreservative for storage of HAM for grafting in the European Union [47]. In this context, had dispase or thermolysin been effective denuding agents, they could represent regulatory issues, since the residual activity of these proteinases may not be easily neutralized.

Using gentle rubbing of HAM with 0.25–0.5 M NaOH-soaked cotton tip, we were able to nearly completely denude fresh or DMSO-cryopreserved membranes within 1 min without any apparent damage to the BM. In some instances a slightly longer time was needed to remove all cells, which may be due to physico-chemical heterogeneity of HAM depending on the distance from placental disc [54]. Additionally, since HAM is human donor material, some donor-to-donor variability could be expected to marginally affect the denuding time.

Staining patterns of the major HAM components were further examined by immunostaining. Generally, the composition of HAM resembled that of limbal epithelial BM, with some differences. Both structures showed continuous staining for laminin α2 and γ3 chains, and type IV collagen α1/α2 chains that are not revealed in the central corneal epithelial BM [55]–[57], as well as for other components present in both central corneal and limbal epithelial BM, such as type IV collagen α6 chain, laminin γ1 and γ2 chains, nidogen-2, perlecan, and fibronectin (see Figs. 3–5). Contrary to a recent study [17], we were unable to obtain positive HAM staining for laminin β1 and β2 chains, but did find laminin α2 and γ3 chains. These discrepancies could arise from different antibodies and fixation protocols used.

Various treatments tested did not show any significant alterations in the patterns of HAM components. In particular, after NaOH denuding, there was no change in continuity and strength of staining for any component compared to the untreated membranes (Figs. 3–5). One can conclude that brief exposure of the epithelial side of HAM to mild NaOH solutions did not adversely affect its structure. Further proof of NaOH usability was obtained upon seeding of various cells on denuded HAM. All cell types including primary LESC and iPSC grew well on NaOH-denuded HAM and formed monolayers in one-two weeks. Over more than a year, no batch of NaOH-treated HAM showed any adverse effect on cell spreading or growth. Primary limbal cultures on this denuded HAM were positive for several putative LESC markers (Fig. 8) with patterns typical for these cells.

Overall, the new method of denuding HAM by brief exposure of its epithelial side to mild sodium hydroxide solution proved to be fast, easy, and reproducible. NaOH decellularization does not alter normal patterns of major HAM components and supports growth of various cell types including LESC. The NaOH treatment also works well on glycerol-cryopreserved HAM, where other methods failed to provide efficient deepithelialization. Because of these advantages, this method may be easily standardized and could become a treatment of choice for preparing HAM in order to propagate LESC under good manufacturing practice (GMP) protocol for clinical transplantation treatment of patients with LSCD.

## Materials and Methods

### Ethics Statement

Human placentas were obtained, after written informed consent from the prospective mothers, upon their Cesarean-section deliveries (approved IRB protocol Pro00019230, part of IRB protocol #9313 for Cedars-Sinai Medical Center, and approved by State Institute for Drug Controls for Charles University). Primary LESC cultures were obtained from donor corneoscleral rims discarded after penetrating keratoplasty. Work with these rims including culturing LESC and iPSC generation was fully covered by an approved Cedars-Sinai Medical Center IRB protocol Pro00019393.

### Preparation of Denuded Human Amniotic Membrane (HAM)

The placental tissue was placed in low glucose Dulbecco’s Modified Eagle’s medium (DMEM; Invitrogen/Life Technologies, Carlsbad, CA, cat# 11885-084 or Sigma-Aldrich, St. Louis, MO, cat# D5546) with antibiotic/antimycotic solution (Invitrogen, cat# 15240062 or Sigma-Aldrich, cat# A5955). The placenta was washed first with Hanks’ balanced salt solution (HBSS, Life Technologies, cat# 24020117) or phosphate-buffered saline (PBS; Life Technologies, cat# 10010049) to remove excess blood. Then the amniotic membrane was mechanically separated from the chorion with forceps and washed three times with HBSS or PBS to allow the spongy stromal side layer to swell, which was then removed. The HAM was cut into 5×5 cm and cryopreserved in PBS with 10% dimethyl sulfoxide (DMSO; Sigma-Aldrich, cat# 2438). In some experiments, HAM was cryopreserved with 50% glycerol, which is a mild fixative [58] and a common cryoprotectant approved in the European Union for HAM cryopreservation [47]. In these cases HAM (3×3 cm) was placed on Sanatyl 20 support (warp knitted 100% polyester fiber bandage, Tylex, Letovice, Czech Republic) and immersed in 50% DMEM with antibiotic/antimycotic solution, 10% fetal bovine serum (Life Technologies, cat# 26140079), 0.38% sodium bicarbonate (Life Technologies, cat# 25080094), and 50% glycerol (Sigma-Aldrich, cat# G2025). Cryopreserved HAM was stored at −80°C until use. After thawing and sequential washing in PBS for 10–30 min at room temperature to remove DMSO or glycerol, HAM was de-epithelialized using different methods for comparison as follows:

1. EDTA treatment. HAM was incubated in 0.02% EDTA (Sigma-Aldrich, cat# E-5134) in calcium-magnesium free PBS at 37°C for one hour to loosen amniotic epithelial cells, and then transferred into PBS. Treatment of membranes was followed by immediately washing twice for 15 min in PBS to remove cellular debris.
2. EDTA-scraping treatment. EDTA-treated HAM was subjected to gentle mechanical scraping with an electric toothbrush on low speed and then washed twice for 15 min in PBS to remove cellular debris.
3. EDTA-heptanol treatment. EDTA-treated HAM was placed in CellCrown™ inserts (Sigma-Aldrich, cat#Z681792 for 6 well plate insert or cat# Z681830 for 12 well plate insert) with epithelial side facing up and was rubbed for 1–2 min with cotton-tipped applicators (Cardinal Health, Dublin, OH, cat# C15053-006) soaked in n-heptanol (1-heptanol, Sigma-Aldrich, cat# 72954-5ML-F). HAM was then washed twice for 15 min in PBS.
4. Thermolysin treatment. HAM was incubated in a 125 µg/ml thermolysin (Sigma-Aldrich, cat# T7902) solution in PBS for 9 min at 37°C as described previously [39], [40]. Treated membranes were subjected to gentle mechanical scraping and then rinsed and washed three times for 15 min in PBS to remove cellular debris or immediately rinsed and washed three times for 15 min in PBS with shaking to remove cellular debris.
5. NaOH treatment. HAM placed in CellCrown™ inserts (Sigma-Aldrich) with epithelial side facing up was de-epithelialized by rubbing with cotton-tipped applicator soaked in 0.5 M NaOH (Sigma-Aldrich, cat# S5881) for 20–30 seconds and followed by immediate 10–15 min washing in HBSS or PBS. In later experiments, 0.25 M NaOH solution was used with additional gentle rubbing using a cotton-tipped applicator.

HAM pictures were taken with Olympus CKX41 inverted microscope equipped with an Olympus C-3040 camera (Olympus, Tokyo, Japan). In some experiments, glycerol-cryopreserved cells were stained with 0.2% TB (Sigma-Aldrich, cat# T6146) in PBS for 90 sec, followed by rinsing 3–5 times in PBS. Because cells are dead after cryopreservation in glycerol [43], they all stained with TB, which made it easy to monitor their further removal.

### Immunostaining

Different types of denuded HAMs from 2–4 independent treatment experiments were embedded for immunocytochemistry in Tissue-Tek® O.C.T. compound (VWR, Visalia, CA, cat# 25608-930). Well-characterized antibodies to BM components (Table 1) and putative LESC markers were used for indirect immunofluorescence as described [44], [56].

**Table 1:** Antibodies used in the study.

Antigen	Antibody	Source	Dilution
Fibronectin	Mouse mAb 568	Ref. [56]	1∶60
α1/α2 type IV collagen	Mouse mAb M3F7	Developmental Hybridoma Bank	straight
α6 type IV collagen	Rat mAb H63	Ref. [56]	straight
Laminin α2	Mouse mAb 1F9	Ref. [56]	straight
Laminin β1	Rat mAb LT3	Ref. [56]	straight
Laminin β2	Mouse mAb C4	Developmental Hybridoma Bank	straight
Laminin γ1	Rat mAb A5	Ref. [56]	straight
Laminin γ2	Mouse mAb MAB19562	Millipore	1∶50
Laminin γ3	Rabbit pAb R96	Ref. [44]	1∶200
Perlecan	Rat mAb C11L6	Ref. [64]	straight
Nidogen-2	Rabbit pAb 1080	Ref. [56]	1∶200
Keratin 14	Mouse mAb sc-53253	Santa Cruz Biotechnology	1∶10
Keratin 15	Mouse mAb sc-47697	Santa Cruz Biotechnology	1∶10
ΔNp63	Goat pAb sc-8609	Santa Cruz Biotechnology	1∶20
PAX6	Rabbit pAb PRB-278P	Covance	1∶100

mAb, monoclonal antibody; pAb, polyclonal antibody.

### Cell Culture on NaOH-denuded HAM

NaOH-treated HAM was used to culture telomerase-immortalized human corneal epithelial cell line (tHCEC), primary human LESC or iPS cells derived from human LESC or skin fibroblasts. Primary LESC-enriched cultures were prepared from discard corneoscleral rims from healthy donors, obtained from collaborating surgeons under an approved IRB protocol CR00004366. Cells were isolated by the dispase method. Corneoscleral rims with conjunctiva cut out with scissors were treated with 2.4 U/mL Dispase II (Roche Applied Science, Indianapolis, IN, cat# 04942078001) in keratinocyte serum-free medium (KSFM; Life Technologies, cat# 17005042) supplemented with 10% FBS at 37°C for 2 hrs. [59]. The limbal epithelial cells eased off the rims were dissociated with 0.25% trypsin (Life Technologies, cat# 15050065) for 30 min at room temperature. Cells were first seeded on a mixture of human basement membrane proteins including fibronectin (BD Biosciences, San Jose, CA, cat# 354008), laminin (Sigma-Aldrich, cat# L4445), and type IV collagen (Sigma-Aldrich, cat# C6745-1ML), at 0.5–1 µg/cm^2^, after [60] in KSFM medium. Cells formed confluent monolayers and stained positive for putative LESC markers including PAX6, K14, K15, K17, and ΔNp63. Then they were passaged onto HAM using trypsin and grown in the same medium. LESC-derived induced pluripotent stem cells were obtained using Yamanaka’s non-integrating oriP/EBNA1 (Epstein-Barr nuclear antigen-1)-based episomal plasmid vectors [61], [62] from Addgene (Cambridge, MA). They were first cultured in mTeSR^TM^1 medium (StemCell Technologies, Vancouver, BC, Canada, cat# 05850), gradually changed to LESC Epilife® medium (Life Technologies, cat# MEPI500CA) with a defined growth supplement (Life Technologies, B-27, cat# 17504044 and N-2, cat# 17502048), antibiotic-antimycotic mixture, and human keratinocyte growth supplement (HKGS, cat# S0015; Invitrogen): bovine pituitary extract, 0.2% v/v; insulin, 5 µg/ml; hydrocortisone, 0.18 µg/ml; transferrin, 5 µg/ml; human epidermal growth factor, 0.2 ng/ml. Non-tumorigenic diploid human corneal epithelial cells immortalized by telomerase gene [63] were cultured in EpiLife® medium (Life Technologies) with human corneal growth supplement (HCGS, Invitrogen, cat# S0095) on type IV collagen-coated plates and passaged using 0.05% trypsin (Life Technologies). These cells are somewhat similar to LESC and express putative LESC markers K14, K17, K19, ABCG2, but not K15 or the differentiated corneal keratin K3 (data not shown here). All cultures were incubated at 37°C in a humidified atmosphere containing 5% CO_2_.
